# Computer-Aided Discovery of New Solubility-Enhancing Drug Delivery System

**DOI:** 10.3390/biom10060913

**Published:** 2020-06-16

**Authors:** Mikołaj Mizera, Eugene N. Muratov, Vinicius M. Alves, Alexander Tropsha, Judyta Cielecka-Piontek

**Affiliations:** 1 Department of Pharmacognosy, Faculty of Pharmacy, Poznań University of Medical Sciences, Święcickiego 4, 60-781 Poznań, Poland; mikolajmizera@gmail.com; 2 Laboratory for Molecular Modeling, Division of Chemical Biology and Medicinal Chemistry, UNC Eshelman School of Pharmacy, University of North Carolina, Chapel Hill, NC 27599, USA; murik@email.unc.edu (E.N.M.); alvesv@email.unc.edu (V.M.A.); 3 Department of Pharmaceutical Sciences, Federal University of Paraíba, Joao Pessoa 58059, PB, Brazil

**Keywords:** quantitative structure-property relationship, cyclodextrins, cefuroxime axetil

## Abstract

The poor aqueous solubility of active pharmaceutical ingredients (APIs) places a limit on their therapeutic potential. Cyclodextrins (CDs) have been shown to improve the solubility of APIs, but the magnitude of the improvement depends on the structure of both the CDs and APIs. We have developed quantitative structure–property relationship (QSPR) models that predict the stability of the complexes formed by a popular poorly soluble antibiotic, cefuroxime axetil (CA) and different CDs. We applied this model to five CA–CD systems not included in the modeling set. Two out of three systems predicted to have poor stability and poor CA solubility, and both CA–CD systems predicted to have high stability and high CA solubility were confirmed experimentally. One of the CDs that significantly improved CA solubility, methyl-βCD, is described here for the first time, and we propose this CD as a novel promising excipient. Computational approaches and models developed and validated in this study could help accelerate the development of multifunctional CDs-based formulations.

## 1. Introduction

Poor solubility of active pharmaceutical substances (API) places a significant limitation on their clinical use. This issue affects nearly 40% of currently marketed APIs, resulting in their low bioavailability and the necessity of increased API doses. Unfortunately, this issue is persistent: it has been estimated that 90% of APIs currently under development are poorly soluble [1,2]. APIs with low solubility belong to groups II and IV in the Biopharmaceutics Classification System (BCS). BCS II substances are poorly soluble and have high permeability, whereas group IV substances are poorly soluble and have low permeability. Optimizing pharmaceutical formulations toward greater solubility may substantially improve their bioavailability.

The use of solubilizers as excipients allows for a compromise between increasing the concentration of APIs at the site of release and maintaining the lipophilic nature of the APIs that facilitates their permeability [3]. Among many strategies for combating poor solubility, the use of solubilizing excipients such as cyclodextrins (CDs) is one of the most popular ones. CDs are macrocyclic polymers that can encapsulate API molecules within the lipophilic cavity or adhere to API at the hydrophilic surface. CDs have been successfully used as solubilizers for many sparingly soluble APIs [4,5,6]. Multiple examples have demonstrated the plausible effect of CDs on the chemical stability of APIs [7], including the pronounced stabilizing effect on β-lactam antibiotics in an acidic environment [8]. The stabilizing effect of CDs on the crystal structure [9] is crucial for preserving the required dissolution rate throughout the shelf period of drugs, such as cefuroxime axetil (CA) [10]. The separation of diastereomers, affecting the bioactivity of CA, may be achieved by the application of CDs [11]. CDs as multifunctional excipients may be also used for taste masking [12,13,14], which is especially important to improve the comfort of patients taking bitter drugs such as β-lactam antibiotics.

The vast number of experimental studies on CDs as multifunctional excipients has contributed to the availability of large data sets with known thermodynamic properties of API-CD systems, including synthetic CDs approved by FDA/EMA for use in pharmaceutical development or as food additives. Indeed, many poorly soluble APIs have entered the pharmaceutical market as a result of using CDs for drug formulations [15]. Many observations indicating that the solubilizing effect of CDs on APIs depends on the structures of both components have made the API–CD systems a good target for quantitative structure–property relationship (QSPR) modeling. If successful, such models could be very useful in the rational design of effective formulations.

The interaction of APIs with CDs can be characterized by the stability constant *K_s_* [7], which is related to change of API solubility Δ*S* according to the formula:

$$\Delta S=\frac{K_{s}S_{0}}{1+K_{s}S_{0}}\left[CD\right]$$

where:

*S*_0_—intrinsic solubility of API,
*K_s_*—stability constant of API-CD system,
[*CD*]—CD concentration.

Machine learning-based QSPR models have shown good performance in predicting *K_s_*. For instance, Jeschke et al. [16] developed a model for *K_s_* prediction of βCD systems. Their model employed data on consistently measured ΔG of formation for βCD systems, and the random forest method achieving the external validation R^2^ = 0.66. Zhao et al. [17] compared gradient boosting and deep neural network models for predicting ΔG of complex formation. Gradient boosting showed good predictive performance indicated by consistent in-sample and out-of-sample scores R^2^ = 0.86, while DNN resulted in a model with R^2^ = 0.76 and R^2^ = 0.62 for in-sample and out-of-sample predictions, respectively. A computer-aided study resulting in the synthesis of a novel polymer drug carrier system was performed by Alves et al. [18]. The model used descriptors of both API and polymer to streamline the selection of the most promising carrier system for poorly soluble APIs. The predictions were experimentally validated and led to the discovery of a new solubility enhancing carrier system.

Herein, we have investigated the use of optimal CD to improve the solubility and bioavailability of CA, a sparingly soluble β-lactam analog. Although the increased solubility of CA in an amorphous form was shown [19], some limitations related to recrystallization during shelf time apply. The possible application of CDs to stabilize the amorphous state of solid CA may be beneficial for achieving higher stability and better solubility [20]. Furthermore, improved compliance may be achieved by masking the extremely bitter taste of CA [21].

In the previous studies, we investigated the benefits of combining CA with CD in the solid phase in terms of improving CA’s solubility and antimicrobial activity [22]. Herein, we have carried out the QSPR modeling of *K_s_*. The main objective of this study was computer-assisted identification of stable CA–CD systems with increased solubility of CA. To achieve this goal, we executed the following specific studies: (i) collection, curation and integration of publicly available data on the stability of CD complexes with small molecules; (ii) development of novel descriptors of API–CD systems; (iii) QSPR model development and validation, (iv) prediction of ln(*K_s_*) for novel CA–CDs complexes for CDs in our in-house collection and (v) experimental validation of computational predictions.

## 2. Materials and Methods

### 2.1. Data Integration and Curation

Data used in the study were acquired from the Cyclodextrin knowledgebase [23]. The data reported in the database contained the IUPAC name of API, IUPAC name of CD, the value of the stability constant and the experimental conditions of stability constant evaluation if reported in the source study. The structure of molecules was generated using OPSIN [24] and ChemAxon [25] IUPAC name converters, and subsequently standardized using ChemAxon Standardizer [26] following the protocols we developed previously [27,28]. Chirality information was preserved during structure generation from the IUPAC names. The curated dataset is available as supplemental material (File S1).

### 2.2. Molecular Descriptors

Descriptors for APIs structures were calculated using Mordred [29], which implemented PaDEL descriptors in Python. 3D descriptors were used, since chirality information was present in provided structures. A total of 637 descriptors were successfully generated and used for model development. A novel protocol for calculating CD descriptors was developed. The main macrocyclic ring was described by a single number of glucose subunits and concatenated with Mordred descriptors of the C-5 side chain terminated by a hydrogen atom. This procedure allowed for the increased variance in descriptor space due to the separation of a similar macrocyclic ring from side chains.

### 2.3. Model Development and Validation

The gradient boosting algorithm was used to create the QSPR model. The basis of operation of the algorithm is an iterative addition of decision trees to an ensemble to reduce the error of the previous trees in this ensemble. The specific implementation used in our modelling study was LightGBM library [30]. The model was validated using a 5-fold external cross-validation procedure [31]. The procedure involves a random division of the entire dataset into five subsets of nearly equal size followed by the systematic binary division of the original dataset into a training set with 80% of all samples used for model training and 20% of the samples used as a test set, such that each of the five subsets would be used once as a test set. For each 80:20 division, a new model was trained and used for the prediction of the test set. The test predictions were collected and used for statistical characteristics calculation. For the final model, a 5-fold cross-validation grid-search was done on the whole dataset, and the best model was refitted on the whole dataset before predicting ln(*K_s_*) of the CA–CD systems.

### 2.4. Statistical Analysis

Predictive performance evaluation of the model was done by calculation of the following measures: accuracy, area under the receiver operating characteristic curve (AUC), correct classification rate (CCR), sensitivity, positive predictive value (PPV), specificity and negative predictive value (NPV) [18].

### 2.5. Systems Stability Prediction and Experimental Evaluation

A poorly soluble API from the BCS II class, CA was selected as a model compound for which the best CD expected to improve CA solubility was predicted. The CD library consisted of an in-house collection of CDs commonly used in pharmaceutical formulations, including αCD, βCD, HP-αCD, HP-βCD and methyl-βCD. For each CA-CD system, predictions were evaluated experimentally. Stability constants of CA-CD systems were measured using the phase-solubility technique [6]. The excess of CA was added to a 15 mL of CD solutions in concentrations of 0.02–0.1 mmol in stoppered test tubes. The test tubes were shaken on a rotary shaker for 72 h at a controlled temperature 25 °C and pH = 7. The samples were filtered by a 25 μm filter and the concentration of CA was measured using UV spectroscopy at 281 nm with the PerkinElmer Lambda 35 UV/Vis Spectrometer.

## 3. Results

### 3.1. Study Design

**Figure 1:**
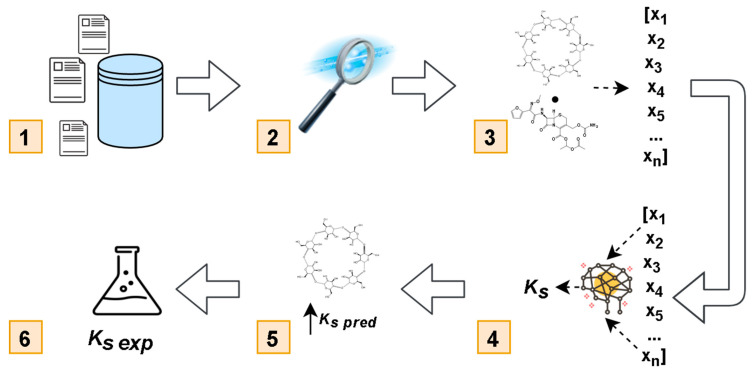
Study design: data collection (1); data curation (2); descriptors calculation (3); model development (4); ln(*K_s_*) prediction (5); experimental validation (6).

The study design is shown in Figure 1. Data on the stability of multiple small molecules-CD systems characterized by their *K_s_* were collected from publicly accessible sources, as described in detail in Methods (Figure 1, Step 1). The data were curated (Figure 1, Step 2), and the reported experimental values were converted to ln(*K_s_*). Small molecule-CD systems were characterized by standard descriptors and a novel protocol for CD descriptor generation was used (Figure 1, Step 3). QSPR models were developed with a gradient boosting machine learning approach (Figure 1, Step 4). Models were employed to predict the stability of CA–CD systems for five CDs from our in-house collection, as described in Methods (Figure 1, Step 5), and all five systems were tested experimentally (Figure 1, Step 6).

### 3.2. Dataset Preparation

**Figure 2:**
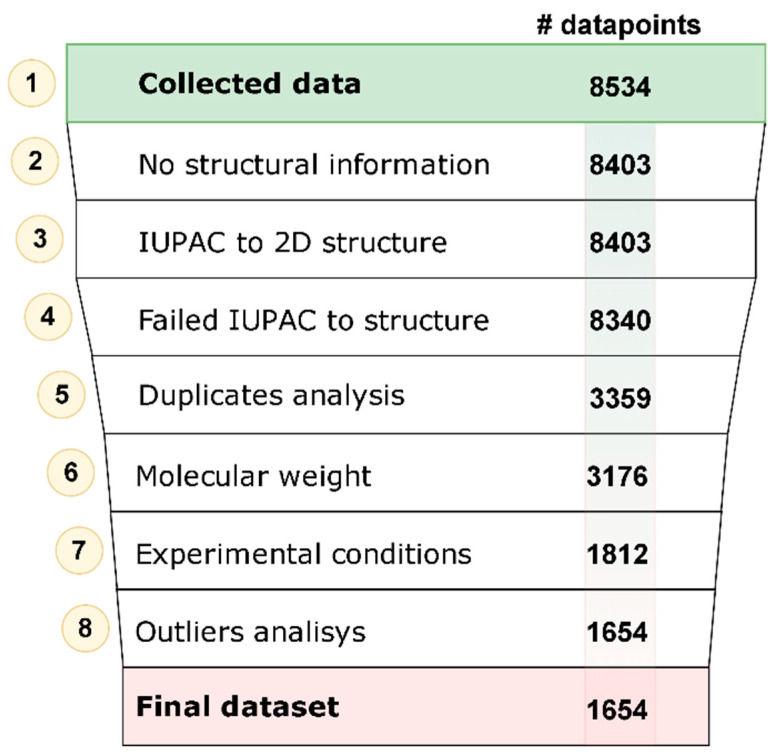
Data curation workflow. Number of data points shown in each row represent the number of compounds left after the respective curation step.

We employed data reported in the database of CD complexes with small molecules [23] that initially included 8534 records (Figure 2, Step 1). These data were curated based on the protocols described by us earlier [27,28]. Records without structural information (131 in total) were removed (Figure 2, Step 2). These included records for compounds for which neither SMILES nor IUPAC names were provided. For the remaining compounds, their IUPAC names were converted to 2D chemical structures by ChemAxon software [25] (Figure 2, Step 3) or manually in the case of failure of the automated procedure. Sixty-three records with IUPAC names that could not be associated with molecular structure were removed along with single-atom counter-ions (Figure 2, Step 4). All records included data on the experimental conditions such as the temperature and pH; for records with missing experimental conditions, the normal conditions, i.e., T = 25 °C and pH = 7, were assumed. Records in the dataset were considered duplicative if both InChI keys of their small molecules and corresponding CDs in the system, as well as experimental conditions, were identical. If the difference between ln(*K_s_*) values for duplicate small molecule-CD systems under the same experimental conditions was less than 10% of the range of ln(*K_s_*) values, duplicative records were merged, and the averaged ln(*K_s_*) value was assigned to the merged record; otherwise, all duplicated records were removed (Figure 2, Step 5). This step resulted in 418 merged records and 4540 records that were removed. The structures of small molecules with the molecular weight greater than 50 Da and less than 500 Da were kept (Figure 2, Step 6). The execution of Step 6 resulted in removing 183 records.

Records with reported temperature and pH close to our test conditions T = 25 °C and pH = 7 were kept. Therefore, we selected only data from the experiments carried out under temperatures between 20 °C and 30 °C and pH between 5 and 8 (Figure 2, Step 7). The final dataset included 1654 small molecule-CD complexes with ln(*K_s_*) values ranging from ln(*K_s_*) = 2 to ln(*K_s_*) = 10, where the datapoints outside these boundaries were considered outliers (Figure 2, Step 8). The distribution of CD types in the final dataset is shown in Table 1. The dataset is dominated by natural CDs: α, β and γ. Of the underrepresented class of semisynthetic CDs, the representatives with the highest counts were hydroxypropyl-βCD (HP-βCD) and methylated derivatives of βCDs (Dimethyl-βCD, randomly methylated-βCD, Trimethyl-βCD). The entire dataset is included in the Supplemental Materials (S1).

### 3.3. Model Development

**Figure 3:**
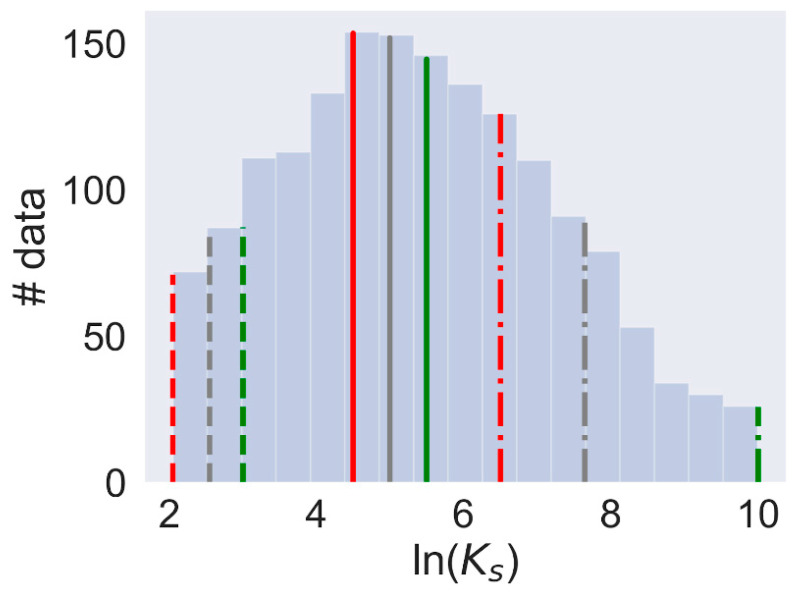
Distribution of ln(*K_s_*) values in the dataset. Thresholds for binary classification models: 4.5 (red), 5.0 (gray), and 5.5 (green).

The data used for model development showed normal distribution with the mean ln(*K_s_*) = 5.37 and standard deviation = 2.22 (Figure 3). The regression model we built initially failed to achieve acceptable statistical characteristics. A possible cause for the failure was the relatively large fraction of the data with missing experimental conditions and the inadequacy of our guessed conditions for building a successful regression model. Therefore, we decided to switch from the regression approach to the more approximate binary classification. The final model consisted of three binary models developed for three different binary division thresholds of ln(*K_s_*): 4.5 (Figure 3, red), 5 (Figure 3, gray) and 5.5 (Figure 3, green). The selection of thresholds for different binary classifications was done based on the range of reported stability constants of ln(*K_s_*) for CA–CD systems [32,33]. For negative classes, the values within 1.5 logarithmic units below the chosen threshold were used (Figure 3, dotted lines). The positive values included data above the threshold; the exact upper boundary was adjusted to achieve an approximate match between the number of positive samples and that of the negative samples. The classification dataset with the threshold of 4.5 included a total of 1145 samples, with 584 positives and 561 negatives (Figure 3, red); for classification with the threshold 5, the dataset included a total of 1316 samples, comprising 671 positives and 645 negatives (Figure 3, gray); and for classification with threshold 5.5, the dataset included 1460 samples: 745 positives and 715 negatives (Figure 3, green). Models were trained and tested using the five-fold external cross-validation procedure, and the statistical characteristics of the models are given in Table 2. The model achieved acceptable scores for all main statistical characteristics according to the suggested best practices for QSAR models validation [34].

### 3.4. Prediction and Experimental Validation

Model-based predictions in comparison with the experimental results are summarized in Table 3. Systems were considered promising if all three models predicted a CD–CA system as positive. According to this protocol, CA–βCD and CA–methyl-βCD were selected as promising hits, and all classification predictions for these two systems were confirmed experimentally (Table 3, Systems 2 and 5). The CA–βCD system was investigated by Sapte et al. [33] before our study, and ln(*K_s_*) = 5.83 was reported for this system. However, we decided to exclude this record from our training database, and use it for an additional external validation of both the experimental data collected in our laboratory and the model we developed. Indeed, the CA–βCD system was predicted correctly as highly stable, and our experimental value of ln(*K_s_*) = 5.72 also agreed with the literature data. To examine the predictive performance of the models further, the three systems selected as not promising (Table 3, Systems 1, 3 and 4) were also tested. The experimental results for these systems were in full agreement with predictions for CA–αCD and CA–HP-αCD and in partial agreement for CA–HP-βCD. The latter system showed high ln(*K_s_*), which was correctly predicted by two out of three models. In total, out of 15 predictions for all tested systems, 14 (93.3%) were correct; i.e., the ln(*K_s_*) value for the CA-HP-βCD system was underestimated in one case (Table 3, System 4). Results obtained in our laboratory were in agreement with the value ln(*K_s_*) = 5.95 reported by Shah et al. [32] (Table 3, System 4) confirming the compatibility of our measurements with the data reported in the independent literature.

## 4. Discussion

The first goal of the study was to collect and curate data required for the machine-learning-based QSPR model. The curated dataset consisted of diverse API and non-API chemical structures in systems with 16 CDs of natural and semi-synthetic origin. Previously, to model guest-CD systems, an effort was made to curate the data originating from the distinct literature studies [35,36], including a large library of both guest molecules and CDs [17]. We have followed the best practices of QSAR modeling [31] to perform our study and assure of its reproducibility.

Initially, we developed continuous QSAR models that failed to achieve acceptable statistical characteristics, i.e., the Q^2^ was <0.5. Thus, we decided to employ binary classification models and developed three models with cut-off thresholds of 4.5, 5.0 and 5.5, respectively. To develop these models, we employed the gradient boosting algorithm and chemical descriptors reflecting the structure of both APIs and side chains of CDs. The size of the CD macrocycles was described by the number of constituting glucose residues. Due to the enantioselectivity of CDs [37], the information on API chiral centers was preserved by using 3D descriptors. One of the biggest advantages of this model is that it could be applied to a variety of CDs and experimental conditions instead of being specific for a single CD and predefined temperature and pH [35,38]. Consideration of both guest and CD structures makes the model useful in cases when no sufficient experimental data for a CD system exist. This problem is especially common when synthetic derivatives of CDs are used. The predictive power of all the models was rigorously validated using 5-fold external cross-validation (see Table 2 for statistical characteristics).

To achieve the goal of this study, we have applied the developed QSPR models to the prediction of the stability constant for a poorly soluble β-lactam antibiotic, CA, combined with five CDs from our in-house collection. We were particularly interested in combinations that would be predicted as positives by all three models as an indication of the higher confidence in the positive result. For all tested systems, 14 out of 15 predictions were correct. The predictions were used to identify the system with likely the highest ln(*K_s_*). Two CA-CD systems (2 and 5 from Table 3) were selected as the most promising, because the models predicted these systems to have high ln(*K_s_*) values; three other systems were expected to have lower ln(*K_s_*) values. Both systems predicted to have high ln(*K_s_*) were confirmed successfully with a minor exception for the CA-HP-βCD system (4 in Table 3), and correct predictions were made by two out of three models. One of the models with the highest ln(*K_s_*) threshold for binary division of the training set of ln(*K_s_*) = 5.5 mis-predicted this compound as negative, whereas this system possessed the highest experimental ln(*K_s_*) values. As the most important outcome of our study, the identified CA–methyl-βCD (5 in Table 3) is a novel, not previously reported CD system, which may serve as a promising excipient in drug formulation with CA.

Moreover, CD systems identified in this work may be of additional value for diastereomers separation [11], solid-state chemical stabilization under certain conditions [7] and bitter taste masking [12,13].

## 5. Conclusions

To summarize, we have successfully developed predictive models for assessing the stability of a broad range of API-CD systems. Using these models, we have identified and experimentally confirmed a novel promising CA-methyl-βCD system with improved CA solubility. This success was enabled by the careful study design, including the collection of the expansive and diverse training dataset, thorough data curation and the use of the best practices of QSAR modeling, including rigorous external validation of the models. The successful experimental validation of the developed models using poorly soluble API such as CA proved the applicability of the model to the discovery of novel API-CD systems, where the solubility of the API can be substantially improved by the application of natural and semi-synthetic CDs. The range of this model’s application can be extended to streamlining the choice of CDs for the pharmaceutical analysis of chiral compounds [37,39] and the phytochemical extraction of APIs from plant matrix [40]. In both applications, a high affinity of CDs to the desired molecules may be used to improve the efficiency of the analytical method, or reduce the toxic solvents use.

## Figures and Tables

**Table 1:** Distribution of cyclodextrin (CD) derivatives in the final dataset.

αCD and Derivatives		βCD and Derivatives		γCD and Derivatives	
Type	Samples	Type	Samples	Type	Samples
αCD	411	Acetyl-βCD	19	γCD	160
Carboxyl αCD	1	βCD	638	Hydroxypropyl-γCD	5
Hydroxypropyl-αCD	3	Carboxyl-βCD	15		
Trimethyl-αCD	8	Dimethyl-βCD	52		
		Hydroxypropyl-βCD	136		
		Randomly methylated-βCD	49		
		βCD sulfate	16		
		Sulfobutyl ether βCD	117		
		Succinate-βCD	5		
		Trimethyl-βCD	19		

**Table 2:** Statistical characteristics. The characteristics of the models for 5-fold external cross-validation.

Class	Accuracy	AUC	CCR	Sensitivity	PPV	Specificity	NPV
ln(*K_s_*) ≥ 4.50	0.64	0.69	0.64	0.68	0.63	0.60	0.65
ln(*K_s_*) > 5.00	0.67	0.75	0.67	0.71	0.66	0.64	0.69
ln(*K_s_*) > 5.50	0.70	0.76	0.70	0.69	0.67	0.71	0.72

**Table 3:** Results of experimental validation. Comparison of predicted versus actual stability classes for different cefuroxime axetil (CA)–CD systems and different ln(*K_s_*) thresholds for binary data division. Measured experimental ln(*K_s_*) values are shown in the last column of the table.

API	Cyclodextrin	Predicted ln(*K_s_*) *			Promising System	Experimental ln(*K_s_*)
		>4.5	>5	>5.5		
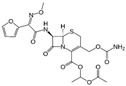 **Cefuroxime Axetil**	^1^ 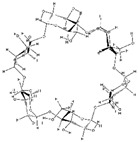 αCD	✓	✓	✓	**No**	4.63
	^2^ 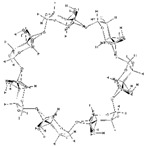 βCD	✓	✓	✓	**Yes**	5.72
	^3^ 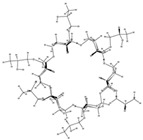 HP-αCD	✓	✓	✓	**No**	4.72
	^4^ 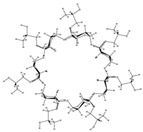 HP-βCD	✓	✓	✕	**No**	5.98
	^5^ 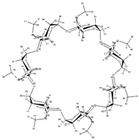 methyl-βCD	✓	✓	✓	**Yes**	5.76

* Accurate prediction is labeled as (✓) while inaccurate is labeled as (✕).

## Supplementary Materials

The following are available online at https://www.mdpi.com/2218-273X/10/6/913/s1, File S1: Dataset used for model development.
